# Impact, Characterization, and Rescue of Pre-mRNA Splicing Mutations in Lysosomal Storage Disorders

**DOI:** 10.3390/genes9020073

**Published:** 2018-02-06

**Authors:** Andrea Dardis, Emanuele Buratti

**Affiliations:** 1 Regional Coordinator Centre for Rare Diseases, Academic Hospital “Santa Maria della Misericordia”, P.le Santa Maria della Misericordia 15, 33100 Udine, Italy; andrea.dardis@asuiud.sanita.fvg.it; 2 International Centre for Genetic Engineering and Biotechnology (ICGEB), Trieste, Italy, I.C.G.E.B., Padriciano 99, 34149 Trieste, Italy

**Keywords:** antisense oligonucleotides, splicing mutations, lysosomal storage diseases

## Abstract

Lysosomal storage disorders (LSDs) represent a group of more than 50 severe metabolic diseases caused by the deficiency of specific lysosomal hydrolases, activators, carriers, or lysosomal integral membrane proteins, leading to the abnormal accumulation of substrates within the lysosomes. Numerous mutations have been described in each disease-causing gene; among them, about 5–19% affect the pre-mRNA splicing process. In the last decade, several strategies to rescue/increase normal splicing of mutated transcripts have been developed and LSDs represent excellent candidates for this type of approach: (i) most of them are inherited in an autosomic recessive manner and patients affected by late-onset (LO) phenotypes often retain a fair amount of residual enzymatic activity; thus, even a small recovery of normal splicing may be beneficial in clinical settings; (ii) most LSDs still lack effective treatments or are currently treated with extremely expensive approaches; (iii) in few LSDs, a single splicing mutation accounts for up to 40–70% of pathogenic alleles. At present, numerous preclinical studies support the feasibility of reverting the pathological phenotype by partially rescuing splicing defects in LSDs. This review provides an overview of the impact of splicing mutations in LSDs and the related therapeutic approaches currently under investigation in these disorders.

## 1. Introduction

**Figure 1:**
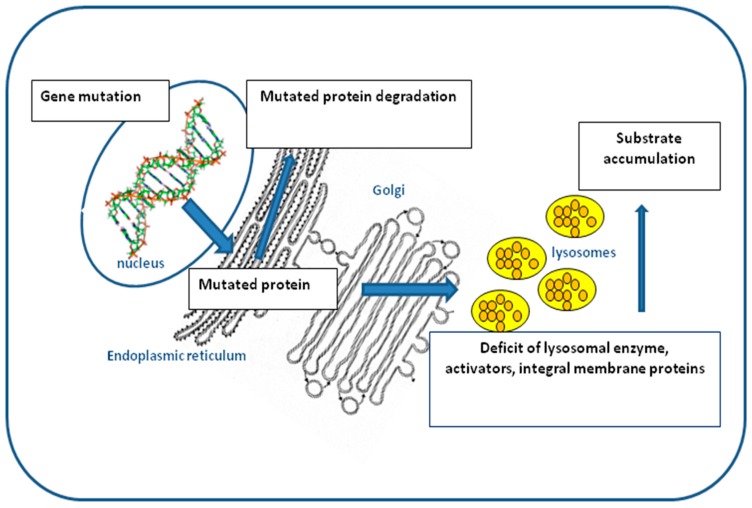
Schematic diagram of the events that lead to lysosomal storage disorders (LSDs). Orange spheres: accumulation of undegraded substrates.

Lysosomal storage disorders (LSDs) are inherited metabolic disorders caused by a deficient function of specific lysosomal enzymes, activators, or integral membrane proteins, leading to an abnormal accumulation of incompletely degraded molecules within the lysosomes [1]. Therefore, LSDs are characterized by intra-lysosomal storage of a variety of substrates—including sphingolipids, glycosaminoglycans, glycoproteins, and glycogen—in multiple tissues and organs (Figure 1).

A mutation in a specific gene leads to the synthesis of a mutated lysosomal protein. Most abnormal proteins are degraded in the endoplasmic reticulum (ERAD). Low levels of the specific enzyme/protein or the presence of a nonfunctional enzyme/protein within the lysosomal compartment cause the accumulation of undegraded substrate.

Clinically, they are all chronic multisystem conditions that present different phenotypes depending on the type of accumulated substrate, the site and the level of accumulation. As a consequence, they are characterized by the variable association of visceral, ocular, hematological, skeletal, and neurological manifestations.

About 50 LSDs have been described to date, with a collective incidence of 1:8000 live births; however, individually they are rare [2]. Most LSDs have an autosomal recessive inheritance, except for Hunter, Fabry, and Danon disease which are X-linked [1].

During the last few decades, enormous progress has been made in the understanding of the molecular basis of LSDs. It is now quite clear that the substrate accumulation triggers a series of secondary cellular effects that eventually lead to cellular death or damage [3]. Each event in this pathogenic cascade represents a potential target for treatment. Indeed, treatment options for some LSDs have rapidly expanded and currently include enzyme replacement therapy (ERT), hematopoietic stem cell transplantation (HSCT), pharmacological chaperone therapy (PCT), gene therapy (GT) and substrate reduction therapy (SRT) [4]. Some of these options are currently under study in clinical trials. However, the primary approved therapeutic approach that has been most successful and broadly used to date is ERT. This approach aims to correct the metabolic defect by the periodic intravenous infusion of a purified or a recombinant lysosomal enzyme. Enzyme replacement therapy has changed the natural history of specific LSDs and have had a huge impact on patient’s quality of life. However, this type of therapy is extremely expensive and treatment of less accessible tissues (such us skeletal muscle, bone, and central nervous system (CNS)) remains a challenge. In particular, due to its high molecular weight the enzyme does not cross the blood brain barrier; therefore, it has no effect in the CNS and patients with neurological features only benefit from the effect of ERT on peripheral tissues. In addition, there is still no specific treatment for many LSDs. For these reasons, there is currently still very much need for additional research to better characterize the most common mutations in responsible genes and to develop novel therapeutic strategies.

To this date, numerous mutations have been described in each gene causing LSDs; among them, about 5–19% affect the pre-mRNA splicing process [5]. In the last decade, several strategies to rescue/increase normal splicing of mutated transcripts have been developed [6] and therapies based on splicing-modifying approaches are swiftly becoming available to patients (as discussed below). From this point of view, LSDs represent excellent candidates for this type of approach for the following reasons: (i) most of them are inherited in an autosomic recessive manner and patients affected by late-onset (LO) phenotypes often retain quite high residual enzymatic activity; thus, even a small recovery of normal splicing may and enzymatic activity may be enough to revert the clinical phenotype; (ii) in few LSDs, single splicing mutations can account for up to 40–70% of pathogenic alleles; (iii) most LSDs still lack effective treatments or are currently treated with extremely expensive approaches.

At present, several preclinical studies support the feasibility of reverting the pathological phenotype by partially rescuing splicing defects in LSDs. This review aims to provide an overview of the impact of splicing mutations in LSDs and the emerging therapeutic approaches based on RNA splicing currently under investigation in these disorders.

## 2. Pre-mRNA Splicing

**Figure 2:**
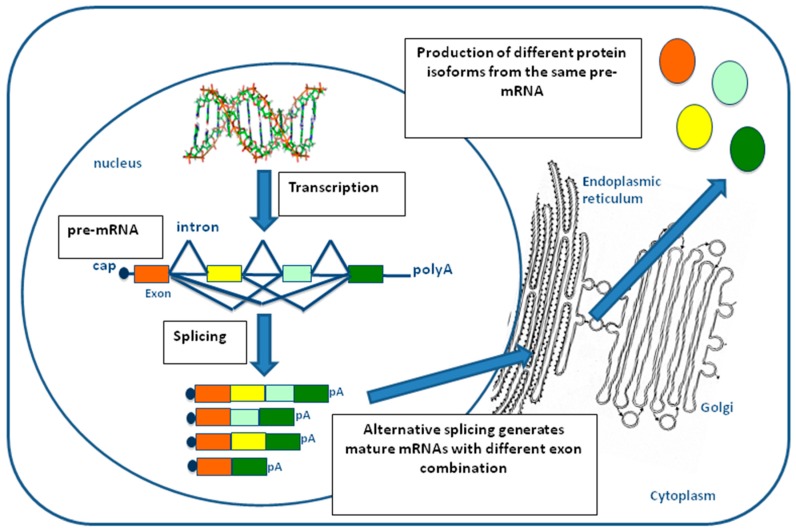
Schematic representation of the splicing process. In pre-mRNA molecules, exons are represented by colored squares and introns by lines. During the alternative splicing process, some exons within a pre-mRNA molecule can be variably included/excluded from the splicing ‘queue’, giving rise to different protein isoforms (colored circles) that can possess distinct biological functions.

In eukaryotes, the nucleotide sequence of most genes encoding for proteins is composed of exons interspersed by introns. Both exons and introns are transcribed into a single pre-mRNA molecule, but only exon sequences are retained during maturation into mRNA through a process of cut and paste that removes the introns and joins together the remaining exons [7]. This process is referred to as constitutive splicing and is catalyzed by a very large molecular machinery, called the spliceosome (Figure 2) [8]. In most human genes, however, one or more exons can be specifically excluded in the mature mRNA depending on specific cell-type, developmental conditions, or following external stimuli [9,10]. This selective choice of exons depending on local context is called alternative splicing and allows the same pre-mRNA molecule to code for different protein isoforms that have distinct and sometimes opposite functional properties [11]. Due to this great flexibility, the process of alternative splicing is the major factor explaining how the approximately 25,000 genes present in humans can produce more than 100,000 proteins with unique amino acid sequences. As can be expected, a better understanding of the regulation of alternative splicing mechanisms, together with the identification and characterization of all the splice variants, still remains one of the greatest challenges in our post-genomic era [12].

In addition to the clear importance of alternative splicing during normal human development, it is now also very clear that, depending on the gene, 15% to 50% of mutations described to occur in human genetic diseases can be associated to alterations at the pre-mRNA splicing process [13]. This makes the pre-mRNA splicing process a highly susceptible pathway to be targeted for the development of novel therapeutic tools that might replace or reduce the need for the expensive traditional approaches.

## 3. Splicing Mutations in LSDs

Currently, at least 600 mutations that affect pre-mRNA-splicing process have been described in patients affected by LSDs [5]. They include mutations that affect consensus 5’ and 3’ splicing sites, (usually located within the 5 nt of the intronic region flanking the exon [14,15,16,17,18]) intronic or exonic mutations that create novel acceptor or donor sites [19,20,21] or intronic variants leading to the pathogenic insertion of pseudoexons within the mature mRNA [22,23]. Most of these mutations are unique or identified in a small number of families, with the following notable exceptions, that present a higher frequency worldwide (acid α-glucosidase gene (*GAA*): c.-32-13T>G mutation; lysosomal acid lipase gene (*LIPA*): c.894G>A) or among specific populations (α-galactosidase A gene (*GLA*): 639+919G>A; *N*-acetylgucosamine-1-phosphotransferase subunits α/β gene (*GNPTAB*): c.2715+1G>A mutation; β-hexosaminidase A gene (*HEXA*): c.459+5A>G mutation).

### 3.1. The c.-32-13T>G Mutation of *GAA* Gene

The *GAA* gene (MIM# 606800), located on chromosome 17q25.2–25.3, encodes the acid α-glucosidase, a lysosomal enzyme involved in the degradation of lysosomal glycogen. Mutations in the *GAA* gene cause glycogen-storage disease type II (GSDII; Pompe disease, acid maltase deficiency, MIM 232300), a neuromuscular LSD characterized by the deficit of α-glucosidase activity, resulting in the lack of glycogen degradation and its progressive accumulation within the lysosomes [24].

Clinically, GSDII is characterized by a highly variable phenotype ranging from a rapidly progressive infantile-onset (IO) to a slowly progressive late-onset form [25]. The classic IO phenotype manifests soon after birth and is characterized by absent or nearly absent enzyme activity, severe muscle weakness, cardiomegaly/cardiomyopathy, and respiratory insufficiency, that typically lead to death within the first year of life [26,27]. Late-onset GSDII comprises all milder subtypes; partial enzyme deficiency manifests in children and adults as a slowly progressive skeletal muscle weakness without cardiac involvement. Respiratory muscle weakness, particularly of the diaphragm, is the leading cause of death in LO cases [26,28,29]. In nearly all patients, the disease progression results in severe physical handicap that heavily affects health status and quality of life [29,30].

To date, 510 different mutations in the *GAA* gene have been identified [5] (with the vast majority of variants being present in single individuals or small number of families. Notably, the only exception is represented by the intronic mutation c.-32-13T>G that is present in 40–70% of the alleles in patients affected with the LO form of GSDII [29,31,32,33,34,35].

The c.-32-13T>G mutation has been described for the first time by Huie et al. in a patient affected by the adult onset form of GSDII [36]. Recently, we have shown that this mutation reduces the binding of splicing factor U2AF 65 kDa subunit (U2AF65) to the polypyrimidine tract of exon 2. This reduction can affect the general efficiency of the splicing process that, in turn, leads to the complete or partial exclusion of exon 2 from the mRNA (known as splicing variants SV2 and SV3, respectively). Since the translational start site is located within exon 2, no protein could be generated from this aberrant transcript. However, it does not completely prevent the expression of the normal spliced transcript (N) and the synthesis of an enzymatically active GAA protein [36,37]. Therefore, patients carrying the c.-32-13T>G mutation, display variable levels of GAA residual activity, that would be enough to delay the phenotypic expression of the disease [38,39].

### 3.2. The c.894G>A Substitution of *LIPA* Gene

The *LIPA* gene (MIM 613497) maps to chromosome 10q23.2–q23.3 and encodes the lysosomal acid lipase (LAL) enzyme, which hydrolyzes cholesteryl esters (CE) and triglycerides (TG) internalized via receptor-mediated endocytosis of plasma lipoprotein particles. Mutations in the *LIPA* gene cause LAL deficiency (OMIM 278000), a clinically heterogeneous LSD. Indeed, homozygous or compound heterozygous mutations leading to a complete lack of LIPA activity cause Wolman disease (WD). This disease is characterized by massive storage of CE and TG in most tissues, vomiting, diarrhea, anemia, failure to thrive, hepatosplenomegaly, adrenal calcification, and death before one year of age [40,41]. Mutations in the *LIPA* gene leading to the synthesis of a LIPA protein that retain residual activity (i.e., 3–8% of controls in blood lymphocytes) cause a less severe disorder known as cholesteryl ester storage disease (CESD) [40,41,42,43,44], characterized by the presence of hepatosteatosis (which may evolve into hepatic fibrosis and micronodular cirrhosis), splenomegaly, mixed hyperlipidemia, hypoalphalipoproteinemia, and premature atherosclerosis [41,42,43,44,45]. Onset of CESD may be diagnosed in childhood or later in life, since its clinical manifestations show a broad spectrum of severity [41,42,43,44,45,46].

At present, 68 mutations have been described in patients affected by LIPA deficiency [5], being the c.894G>A substitution the most frequent mutation among patients affected by CESD. Indeed, this mutation accounts for an estimated 60% of all CESD cases [47]. The observation that the c.894G>A co-segregated with the same *LIPA* gene haplotype in a cohort of Italian patients affected by CESD strongly supports the hypothesis of a common ancestral origin [16]. The mutation, located at position 1 of the 5’ donor site of exon 8 [47], leads to the in-frame exclusion of exon 8 from *LIPA* mRNA, giving rise to the inactive LIPA protein Δ254–277 [48]. However, in vitro experiments using hybrid minigene constructs clearly showed that the c.894G>A substitution resulted in an incomplete exon 8 exclusion, with the production of about 5% of normally processed mRNA, accounting for a variable residual LIPA activity in peripheral blood lymphocytes or fibroblasts ranging from 2% to 12% of the values found in control cells [43,49,50,51,52,53]. Interestingly, a much less frequent severe mutation found in patients affected by WD and involving the same splicing site (c.894+1G>A) resulted in complete exon exclusion.

### 3.3. The 639+919G>A Mutation of *GLA* Gene

The *GLA* gene (MIM 300644), located on chromosome Xq22.1, encodes the lysosomal enzyme α-galactosidase A (α-GalA), which hydrolyses the terminal α-galactosyl of globotriaosylceramide (Gb3) and related glycolipids. α-galactosidase A deficiency, due to mutations in the *GLA* gene causes Fabry disease (FD; OMIM 301500), an X linked LSD characterized by a wide range of phenotypic expression [53]. This disease ranges from a milder phenotype with just cardiac and/or renal abnormalities to classic FD that additionally presents cardiac vascular degeneration, chronic pain, angiokeratoma, and kidney manifestations usually leading to renal failure [54]. Although FD is X-linked, heterozygous females may develop mild to severe clinical manifestations [54,55,56].

The mutational spectrum of *GLA* mutations is very heterogeneous with 911 mutations reported to date [5]. Among the 43 variants reported to affect the splicing process, the c.639+919G>A is associated with the LO cardiac form of FD and is common in the Asian population. Indeed, a newborn screening program of FD in Taiwan, showed that in this population, the incidence of Fabry mutations is 1 in 1368 for males. Approximately 82–86% of them carry the c.639+919G>A mutation [57,58].

The *GLA* gene transcribes two alternatively spliced mRNAs: the most abundant one contains the 7 *GLA* exons and encodes α-GalA protein, while the minor one accounts for about 5% of total *GLA* transcripts [23,59] and retains a 57-nt-long cryptic exon located within intron 4. This later transcript is predicted to encode for a shorter protein of unknown function [23]. The c.639+919G>A mutation, located 4bp upstream of the 3’ splice site of the cryptic exon, increases the recognition of a normally weak splice site, causing a remarkable imbalance in the expression of the two *GLA* transcripts, with an increase in the alternatively spliced *GLA* mRNA and a significant reduction of normal spliced mRNA, which would account for the 5–10% residual α-GalA activity detected in patients with the cardiac LO form of FD [23].

### 3.4. The c.2715+1G>A Mutation of the *GNPTAB* Gene

The *GNPTAB* gene (MIM 607840) encodes the α/β subunits of the *N*-acetylgucosamine-1-phosphotransferase, α2β2γ2 hexameric complex responsible for the initial step in the synthesis of the mannose 6-phosphate (M6P) recognition markers on lysosomal enzymes in the Golgi apparatus [60]. Failure to attach this recognition signal leads to the mistargeting of all lysosomal enzymes that require the M6P marker to enter the lysosome.

Mutations in the *GNPTAB* gene leads to Mucolipidosis types II (MLII or I-cell disease; MIM 252500) and III α/β (MLIII α/β, or pseudo-Hurler polydystrophy; OMIM 252600), an autosomal recessive disorder characterized by the intracellular deficiency of multiple lysosomal enzymes and very high enzyme activity in extracellular fluids such as plasma and serum.

Clinically, Mucolipidosis type II is characterized by the early onset of symptoms that include severe developmental delay, generalized hypotonia, gingival hyperplasia, short stature, coarse facial features, and severe radiologic abnormalities. A rapidly progressive clinical course leads to death during the first decade of life. By contrast, Mucolipidosis type III is a much milder disorder with a slower, albeit still progressive, clinical course that generally allows survival to adulthood [60].

To date, 175 mutations have been described in the *GNPTAB* gene, among them 19 affect the splicing process. A recent study performed in a cohort of 16 Chinese patients affected ML II and III α/β showed a high frequency of the splicing mutation c.2715+1G>A, being present in 28% of the alleles [61].

This mutation that leads to the exclusion of exon 13 from the mature *GNPTAB* mRNA has been initially identified in two patients from a Korea [62] and Japan [63], respectively. The exclusion of exon 13 from the mRNA is predicted to cause a shifting in the open reading frame with the consequent generation of a premature termination codon.

### 3.5. The c.459+5A>G Mutation of the *HEXA* Gene

The *HEXA* gene (MIM 606869) encodes the α subunit of β-hexosaminidase A enzyme. Mutations in this gene lead to Tay Sachs disease, an autosomal recessive LSDs characterized by the accumulation of gangliosides (GM2 and GM3) within the lysosomes.

The clinical picture of Tay Sachs disease ranges from the IO of rapidly progressive neurodegenerative form, leading to death before the fourth year of life, to the (LO) form, a progressive neurological condition compatible with survival into childhood (sub-acute form) or adulthood (chronic form) [64].

To date, 183 mutations have been described in the *HEXA* gene, 35 of them affect the splicing process. Although the spectrum of mutations identified in Tay Sachs patients is quite heterogeneous, a high frequency of the c.459+5G>A mutation, was found in Spanish (32.4%) and Argentinian (26.5%) patients affected by the disease [65,66]. Haplotype analysis in the Argentinian cohort suggested that in these families the c.459+5G>A mutation might have arisen by a single mutational event [66].

The c.459+5G>A mutation leads to the exclusion of exon 4 from the mature transcript [67]. The exon 4 skipping would cause a shifting in the open reading frame and the consequent generation of a premature termination codon.

## 4. Therapeutic Approaches for Splicing Defects in LSDs

In the last 10 years, significant advances have been made in the field of RNA based therapies aimed at rescuing splicing of mutated transcripts [68,69,70,71]. Indeed, many kinds of therapeutic approaches are currently under development ranging from the use of small molecules capable of modulating the expression of specific splicing regulatory proteins to the modification of more sophisticated and less well-known splicing pathways such as *trans-*splicing [72]. In the recent years, some of these approaches have been tested using different in vitro models of LSDs with the aim of restoring normal splicing of transcripts carrying both rare and common splicing mutations. In particular, the splicing-therapy approaches mostly used up to now in this group of diseases have included the use of antisense oligonucleotide (AONs) and of modified U1 small nuclear RNAs (U1snRNA) that exploit base-pairing ability to target specific splicing regulatory sequences in the RNAs of interest. Before describing their use for the rescue of LSD-related splicing mutations, however, it is important to describe their general mechanism of action.

The U1snRNA, the ribonucleic acid component of the U1 ribonucleoprotein, mediates recognition of the donor splice site (5′ss) and the first step in the spliceosome assembly [73]. In the mid-80s, Zhuang and Weiner demonstrated that engineered U1 mutated snRNA were able to suppress 5′ss splicing mutation [74]. Subsequently, the ability of modified U1snRNAs with increased complementarity to mutated 5′ss to redirect the spliceosome assembly and to rescue splicing was demonstrated in various cellular [75,76,77,78] and mouse models [79,80] of human genetic disease. This approach has been made possible by the demonstration that eight nt at the single-stranded 5′ terminus of U1 can be replaced by unrelated sequences with up to 50 nt without affecting either stability or the ability to assemble into snRNP particles [81]. Therefore, chimeric U1snRNAs have also been used to express within the backbone of the snRNA sequences that target aberrant splice sites (i.e., containing AONs, see below) [82,83].

Another common RNA modulating therapeutic approach that has been tested in in vitro models LSDs is based on the use of AONs. These short modified nucleic acids are designed to bind a target RNA through complementary base-pairing [84], sterically blocking the binding of abnormal cryptic splice sites or regulatory regions to the RNA binding proteins and modifying the RNA processing. AONs represent a very attractive therapeutic option for several reasons including their low toxicity, deliverability to a wide-range of cells in vivo, and stability, with activity in cells lasting up to a year after a single dose [84,85]. It is worth pointing out that AONS technology [86,87] has already entered clinical trials and recently the American Food and Drug Administration (FDA) has approved this approach for the treatment of Duchenne muscular dystrophy (DMD) [88] and spinal muscular atrophy (SMA) [89]. The complete list of the clinical trials currently involving AONs has been recently reviewed in Havens and Hastings [90]. At present, one of the major challenges in antisense technology is still represented by their delivery in patient tissues [91,92]. However, a number of new technologies are currently being developed to overcome these difficulties (lipid- or polymer-based nanoparticles, antibody- or ligand-linked oligonucleotides, and small molecules that improve delivery) and it is expected that these approaches will considerably speed-up their pre-clinical and clinical development.

Another promising approach is represented by the use of bifunctional oligonucleotides that target a specific sequence within the exon or intron of choice and also carry a tail designed to modify splicing either in a positive or a negative manner [93,94,95,96]. Currently, these bifunctional oligonucleotides have been found to be effective, and as a general rule it has been shown that the effects on exon inclusion mediated by the tails are more affected by changing the number, sequence, and chemistry of the motifs that are coupled to the antisense part of the oligonucleotide rather than the strength of annealing to the target site [97].

### 4.1. In Vitro Studies Using Modified U1snRNA in LSDs

**Figure 3:**
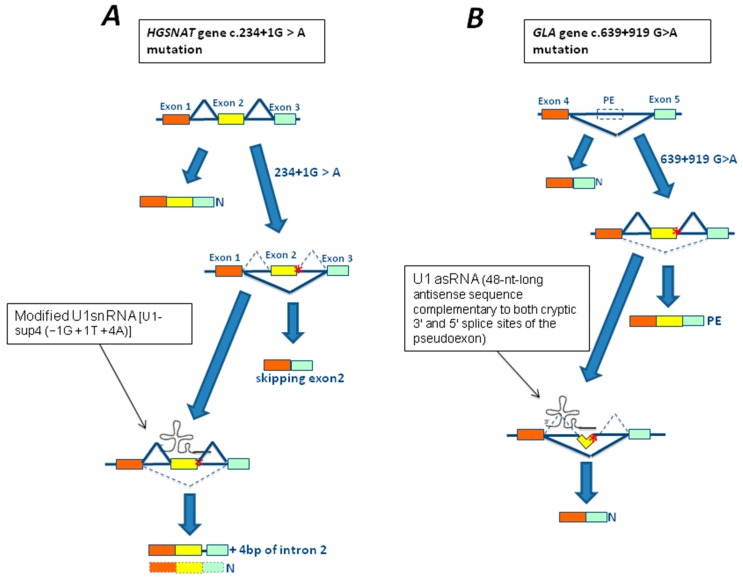
Rescue strategies based on modifying the U1 small nuclear RNAs (U1snRNA) architecture for mutations associated with LSDs. (**A**) Schematic representation of the 5’ portion of the heparan-α-glucosaminide *N*-acetyltransferase gene (*HGSNAT*), where exons are represented by colored rectangles. The c.234+1G>A mutation (red asterisk) leads to the exclusion of exon 2 from the mature mRNA. The use of a U1 fully adapted to the donor splice site of *HGSNAT* exon 2 [U1-sup4 (−1G + 1T + 4A)] resulted in a partial recovery from the splicing defect and the production of another aberrant splicing product which included the first four bp of intron 2. (**B**) Schematic representation of a portion of the α-galactosidase A gene *(GLA*), where exons 4 and 5 are represented by colored rectangles while a pseudoexon is represented by an empty rectangle (PE). The c.639+919G>A mutation (red asterisk) leads to the inclusion of the pseudoexon (yellow rectangle) within the mature mRNA. The use of a chimeric U1 snRNA carrying a 48-nt-long antisense sequence complementary to both cryptic 3′ and 5′ splice sites of the pseudoexon (U1asRNA) resulted in a complete rescue of normal splicing and GLA activity. N: normal spliced mRNA.

Within the LSDs field, an attempt to rescue mutations affecting the 5′ss using modified U1snRNAs with increased complementarity to mutated 5′ss to redirect the spliceosome assembly has been performed by Matos et al. [98] using cellular models of Sanfilippo C disease (or Mucopolysaccharidosis IIIC). In particular, three mutations—c.234+1G>A, c.633+1G>A, and c.1542+4dupA—affecting the 5′ donor site of exons 2, 6, and 15, of the heparan-α-glucosaminide *N*-acetyltransferase (*HGSNAT*) gene respectively, have been studied. Among them, only the treatment of fibroblasts carrying the c.234+1G>A mutation in homozygous state with a U1 fully adapted to the donor splice site of *HGSNAT* exon 2 (U1-sup4 (−1G + 1T + 4A)) resulted in a partial recovery from the splicing defect (Figure 3A). However, sequence analysis of the produced transcript revealed two different sequences: one with the normal splicing; and the other which included the first four bp of intron 2. Unfortunately, no improvement in enzyme activity was observed, suggesting that the partial rescue of the normal transcript was not sufficient to generate a significant improvement in protein production and, in turn, of enzymatic activity [98].

An interesting strategy combining U1snRNA and antisense technologies has been used for in vitro correction of two mutations causing pseudoexon inclusion of the *GLA* gene, the unique c.639+861C>T and the c.639+919G>A highly prevalent in Taiwanese newborns.

This strategy exploited the activity of the U1snRNA promoter to drive the expression of a chimeric U1 snRNA in which nt from position 3–10 at the 5′-end of U1 snRNA, required for the recognition of the 5′ splice site, were substituted with 48-nt-long antisense sequences complementary to both cryptic 3′ and 5′ splice sites of the pseudoexon. Using this approach, it was possible to correct splicing pathways and, most importantly, efficiently restore α-GalA activity in reporter minigenes carrying the c.639+861C>T or c.639+919G>A *GLA* mutations [99] (Figure 3B).

### 4.2. In Vitro Studies Using Antisense Oligonucleotides in LSDs

**Figure 4:**
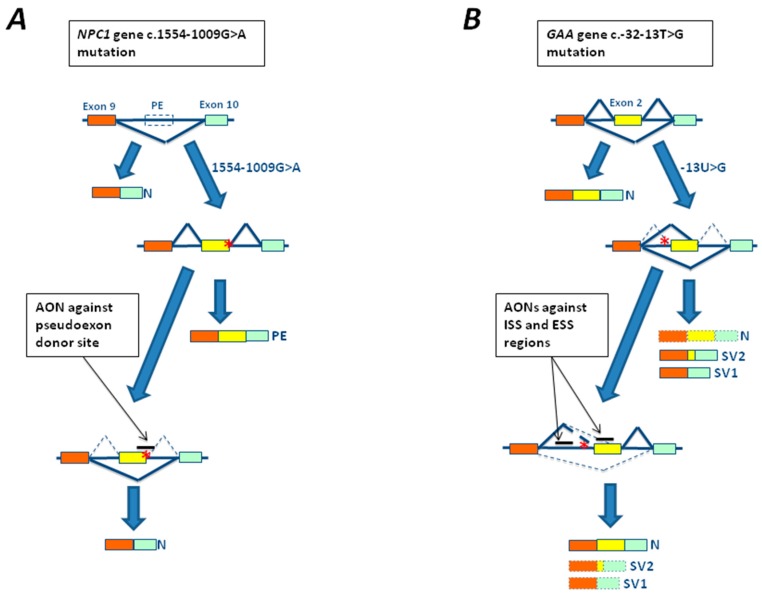
Rescue strategies based on antisense oligonucleotides (AONs) for mutations associated with LSDs. (**A**) Schematic representation of a portion of the Niemann Pick 1 gene (*NPC1*), where exons 9 and 10 are represented by colored rectangles while a pseudoexon is represented by an empty square (PE). The 1554-1009G>A mutation (red asterisk) leads to the creation of a cryptic splice site and the inclusion of a pseudoexon within the mature mRNA (yellow rectangle). The use of a AON targeting the abnormal cryptic splice sites was able to restore normal splicing (N). (**B**) Schematic representation of a portion of the α-glucosidase gene (*GAA*), where exons 1–3 are represented by colored rectangles. The c.-32-13T>G mutation leads to the total (SV1) or partial (SV2) exclusion of exon 2 from the mature mRNA. However, variable amounts of normal spliced mRNA are also generated. The use of AON against splicing silencers sequences identified within exon 2 and intron 1 resulted in a partial rescue of normal splicing and GAA activity above the disease threshold.

The first attempts to develop an AON-based therapy in LSDs were done in cellular models of Niemann Pick C disease carrying the c.1554-1009G>A mutation located in intron 9 of the Niemann Pick 1 gene *(NPC1*) gene identified in a Spanish patient [22]. The mutation creates a new cryptic donor splice site resulting in the incorporation of 194 bp of intron 9 as a pseudoexon in the mature NPC1 RNA (Figure 4A). Transfection of cultured fibroblasts from the patient carrying this mutation with a specific antisense morpholino oligonucleotide targeted to the abnormal cryptic splice was observed to be able to restore normal splicing [22] (Figure 4A).

Recently, important progress has also been made in the development of AON based therapies for the c.-32-13T>G mutation, the most common mutation in the LO form of Pompe disease. Using two different experimental approaches two groups have identified splicing silencers sequences within exon 2 [100] and intron 1 [101,102] of *GAA* gene, respectively. Treatment of both fibroblasts and myotubes (derived from patient’s myocytes or induced pluripotent stem cell (iPSc)) with AONs against these regions resulted in a partial rescue of normal splicing and GAA activity above the disease threshold [100,101,102] (Figure 4B). Most importantly, the use of these AONs against the silencer sequences identified within exon 2 resulted in a significant reduction of glycogen accumulation in patient myotubes [99], providing evidence of the efficacy of this therapeutic approach in reverting the disease phenotype in vitro.

## 5. Open Issues and Conclusions

The field of RNA-based therapies has experienced remarkable progresses in the last few years, with many clinical trials being in an advanced state and the approval of the first antisense drugs becoming available to the market. LSDs represent excellent candidates for this type of approach since patients affected by LO phenotypes usually retain quiet high residual enzymatic activity; thus, even a small recovery of normal splicing may be enough to reach the threshold needed to get a beneficial effect. In addition, in few LSDs, single splicing mutations can account for up to 25–70% of pathogenic alleles; therefore, many patients might benefit from a single approach.

In light of this evidence, it is quite clear that more efforts should be done to identify splicing mutations in LSDs, to better characterize their incidence, and especially to functionally characterize their impact at the molecular level. Clearly, achieving these three goals will be crucially important in order to develop the most appropriate therapeutic strategy for each mutation.

At present, the use of strategies aimed at modifying aberrant spicing patterns has been successfully tested in different models of LSDs. These approaches, although still preliminary, have provided the necessary proof of principle for their use in clinical settings. However, two main issues still hamper the transfer of this technology to the clinics: (i) the lack of appropriate animal models for pre-clinical studies; (ii) the inefficient delivery of the therapeutic agent to target tissues.

Animal models of most LSDs have already been developed. However, they are mostly knock out or transgenic mice carrying null mutations. Therefore, since therapies that target splicing mutations are mutation specific, they are not suitable to in vivo testing this type of approach.

Recently, a murine model of Niemann Pick C (NPC) disease bearing the pseudoexon-generating mutation c.1554-1009G A has been generated. Interestingly, this model recapitulates the main features of NPC disease [103]. Although more effort should be done to develop suitable animal models, this task might not be straightforward due to species differences in the sequences involved in the splicing process.

Second, the effective delivery of RNA based therapeutic agents in vivo remains a major challenge. Indeed, the key problem is to deliver the therapeutic agent to its site (organ or tissue) of action and then into the in cytosol or nucleus of cells within such tissues. However, these relatively small molecules can be coupled to a variety of compounds to improve their delivery and uptake in selected organs. Indeed, a variety of innovative approaches to overcome this issue are currently under development. Among them, the use of high affinity, highly selective ligands that allow a very precise targeting to particular cells or tissues and the use of lipid and polymer-based nanocarriers, represent a very promising field of research. For the treatment of LSDs, however, this may be particularly challenging as multiple organs/tissues will need to be targeted in order to obtain a satisfactory response for the often-complex phenotypes displayed by this kind of disorders. In addition, the use of small molecules that enhance oligonucleotide effectiveness has been proposed. Indeed, endocytosis and intracellular trafficking are mediated by a complex network of proteins, each of them represent a potential target for a small molecule to improve the uptake and trafficking process [91].

In conclusion, about 600 mutations that affect the pre-RNA splicing process have been described in patients affected by LSDs. Few of them are highly frequent among affected patients worldwide or within specific population groups. Several lines of evidence support the in vitro effectiveness of RNA based therapies to rescue aberrant splicing, enzymatic activity, and the pathological phenotype in LSDs. These results are extremely encouraging, in particular considering the lack of therapeutic options the extremely high costs of the available therapies for LSDs. Regarding this last issue, it is worth noting that AONs have a very long shelf-life, a characteristic that considerably lowers their cost of production/storage for health systems. In light of these data, there is an urgent need for the development of suitable animal models and better delivery systems in order to move these approaches to testing in clinical settings.
